# Urinary Acetaminophen Metabolites and Clinical Outcomes in Extremely Premature Infants

**DOI:** 10.1055/a-2512-9387

**Published:** 2025-01-31

**Authors:** Miguel Guardado, Dara Torgerson, Cheryl Chapin, Azuka Atum, Ryan D. Hernandez, Ronald Clyman, Rebecca Simmons, Samuel Parry, Philip L. Ballard

**Affiliations:** 1 Department of Biological and Medical Informatics, University of California, San Francisco, San Francisco, California; 2 Department of Epidemiology and Biostatistics, University of California, San Francisco, San Francisco, California; 3 Department of Pediatrics, University of California, San Francisco, San Francisco, California; 4 Department of Bioengineering and Therapeutic Sciences, University of California San Francisco, San Francisco, California; 5 Department of Pediatrics, Perelman School of Medicine, University of Pennsylvania, Children's Hospital of Philadelphia, Philadelphia, Pennsylvania; 6 Department of Obstetrics and Gynecology, Perelman School of Medicine, University of Pennsylvania, Philadelphia, Pennsylvania

**Keywords:** acetaminophen, paracetamol, metabolomics, extremely premature infant, bronchopulmonary dysplasia, retinopathy of prematurity

## Abstract

**Objective:**

Extremely premature infants are treated with acetaminophen (APAP) for pain and patent ductus arteriosus. High doses of APAP in adults are toxic, and a recent study found an association between APAP metabolite levels in mothers' breast milk and both bronchopulmonary dysplasia (BPD) and retinopathy of prematurity (ROP) in their premature infants. In this study, we determined levels of APAP metabolites in the urine of infants at high risk for BPD and ROP.

**Study Design:**

Biorepository urine samples from 314 infants <29 weeks' gestation in the multicenter TOLSURF and PROP studies were analyzed by untargeted UHPLC:MS/MS (Metabolon, Inc.). We performed multivariate logistic regression and meta-analysis to examine associations between APAP metabolite levels and clinical outcomes.

**Results:**

4-APAP sulfate was the most abundant of eight detected APAP metabolites and was present in 95% of urines. There were high correlations between levels of 4-APAP sulfate and the other APAP metabolites. In longitudinal studies on a subgroup of infants (day 6–56), periods of elevated 4-APAP sulfate occurred in 24/28 infants and were of longer duration (10.5 vs. 4.2 days, *p* = 0.001) with higher levels (13.3 vs. 5.6, *p* = 0.01) in infants after transition to enteral from total parenteral nutrition. Episodes of elevated metabolite did not differ by BPD status. On both days 10 and 28 there were no significant associations between levels of APAP metabolites and either BPD or ROP for all infants or for infants exclusively on parenteral or enteral nutrition.

**Conclusion:**

In two cohorts of extremely premature infants, levels of urinary APAP metabolites were not associated with increased risk for two adverse clinical outcomes.

**Key Points:**

- Safety of acetaminophen (APAP) in extremely premature infants has not been established.
- The major urinary APAP metabolite was detected in the majority of urine samples.
- No association was found between APAP levels and either bronchopulmonary dysplasia or retinopathy of prematurity.

Acetaminophen (paracetamol, APAP), an inhibitor of prostaglandin synthetase, is the most used analgesic worldwide. In the Neonatal Intensive Care Unit (NICU), APAP is used as an opioid-sparing agent for infant pain and discomfort and is also a frontline drug along with indomethacin and ibuprofen for closure of the patent ductus arteriosus (PDA) 1 2 3 In adults, there is an apparent association between APAP dose and hypertension, and APAP overdose is the major cause of liver failure in the US. 4 5 It is also reported that in utero exposure to APAP is associated with increased risk for childhood dysregulated gonadal development, behavioral disorders and asthma. 6 7 8 9 10 11

Concern has been raised regarding the safety of APAP treatment in the NICU, particularly for infants <28 weeks gestational age with developmentally immature lungs and oxidative stress. 12 13 14 Although meta-analyses of clinical trials have found no increased risk of bronchopulmonary dysplasia (BPD) in moderately premature infants treated for PDA with APAP versus nonsteroidal anti-inflammatory drugs, 3 there are observational reports toward increased incidence of BPD in infants (mostly >28 weeks gestational age) exposed to APAP as an analgesic. 15 16 Using a metabolomic approach, Santoro et al recently reported an association between APAP metabolite levels in mothers' breast milk at postnatal days 14 and 28 and the diagnosis of both BPD and retinopathy of prematurity (ROP) in their premature infants. 17

Untargeted metabolomic analysis involves the separation and identification of low molecular weight chemicals by ultrahigh-performance liquid chromatography and mass spectrometry (UHPLC-MS/MS) to provide quantitative data on hundreds of metabolites in a biofluid. Previous studies have examined the metabolome in premature infants using tracheal aspirate/lung lavage fluid, 18 19 20 21 blood, 22 23 and urine, 24 25 26 identifying putative biomarkers for BPD or metabolic responses to treatments; however, there is little metabolome-wide information available for xenobiotic chemicals present in the premature infant.

In this study, we used untargeted metabolomic analysis of biorepository urine samples from two cohorts of premature infants to investigate associations of APAP metabolites with two clinical outcomes (BPD and ROP) that are diagnosed later in the clinical course; we also determined levels of these metabolites over time in a subset of infants to provide profiles of APAP exposure in the NICU. Our findings provide new information on APAP metabolism in premature infants, describe different patterns of exposure, and examine associations between metabolite levels and the two neonatal disorders. A portion of the results have been published as a preprint. 27

## Materials and Methods

### Clinical Cohorts

TOLSURF (Trial of Late Surfactant, ClinicalTrials.gov, identifier: NCT01022580) was a blinded, randomized, sham-controlled trial performed between 2010 and 2013 at 25 US centers to assess the effects of late surfactant treatments on respiratory outcomes. The trial design, infant characteristics, and effects of late surfactant treatment have been reported. 28 A total of 511 extremely low gestational age (<28 weeks) infants who required mechanical ventilation at 7–14 days were enrolled and received either surfactant (calfactant/infasurf) or sham installation every 1–3 days. Clinical information included selected medications (not including APAP), age at full enteral feeds transitioning from total parenteral nutrition (TPN), and respiratory parameters. Urine samples were collected (1–4 per week for up to eight weeks) using cotton balls in the diaper. Incidence of BPD at 36 weeks postmenstrual age, which was defined as a requirement for supplemental oxygen or positive pressure using an oxygen/pressure reduction test, and stage 2 ROP did not differ between treated and control groups.

PROP (Prematurity Respiratory Outcomes Program, NCT01435187) was an observational study performed between 2010 and 2013 at eight US centers and designed to collect clinical data and biospecimens from infants <29 weeks' gestational age for analysis of factors related to respiratory outcomes. Clinical information including selected medications, age at full enteral feeds transitioning from TPN, and respiratory parameters were obtained as in the TOLSURF study. Urine samples were collected at approximately days 7, 14, 21, and 28 using cotton balls in the diaper. BPD was defined as for the TOLSURF study. A total of 835 infants <29 weeks' gestational age were enrolled in PROP at 1–7 days with characteristics as previously described. 29

Plasma was obtained in 2015 from a cohort of 12 newborn premature infants (gestational age 30.6 ± 4.2 weeks, 11 African American and 1 non-hispanic White) and their mothers at the time of uncomplicated premature birth at University of Pennsylvania hospital-affiliated obstetric practices.

The research protocols for all studies were approved by the Institutional Review Boards of the participating institutions, and a parent of each infant provided written informed consent.

### Metabolomic Analysis

Untargeted metabolomic analysis of relative levels of metabolites in urines and plasma by UHPLC-MS/MS was performed by Metabolon Inc (Morrisville, NC) as described. 23 25 26 The samples were analyzed in five separate batches at ≤144 samples per batch: TOLSURF three batches, PROP one batch, and plasma one batch. Quality control procedures included a set of internal standards added to each sample plus pooled urine samples in each batch for adjustment due to instrument variability or batch-to-batch effects. In addition, samples of saline exposed to cotton balls on a diaper (to mimic the procedure used for urine collection) were analyzed as controls; no APAP metabolites were detected. For cross-sectional analysis of chemical levels, metabolomic analyses were performed on a total of 644 urine samples from subsets of infants (171 TOLSURF infants and 143 PROP infants) who had urine samples available at two time points: time point 1 (day 10, range 7–14, median = 10) and time point 2 (day 28, range day 23–30, median = 28). A longitudinal analysis of metabolite levels was performed in 302 urines from 28 TOLSURF infants with ≥5 (mean/sd 11 ± 4) urine samples available over ≥19 (mean/sd 35 ± 8) days; 25 of the 28 infants for the longitudinal study were also included in the cross-sectional study at two-time points. In the time course analyses, we evaluated all metabolite levels and separately those defined as elevated (>2-fold of median).

The urinary metabolite dataset comprised a total of 1435 compounds of both known identity (named by Metabolon as of July 2022, 77%) and unnamed (23%) chemicals. The area under the peak was obtained for each biochemical and adjusted for any inter-assay (batch) variability and urine osmolality and then rescaled setting the median value of all samples for each chemical to 1. For analysis of levels of metabolites between samples, we used these median-normalized values. To compare relative amounts of APAP metabolites in a urine sample, we used area under the peak values and normalized to the value for 4-APAP sulfate, the most abundant APAP metabolite, for each infant. For analysis of nutritional status, we defined full enteral feeds as urine samples collected ≥2 days after discontinuation of all parenteral feeds.

### Statistical Analysis

Univariate comparisons between outcomes and APAP metabolite abundance were analyzed using Fisher's exact test (detected vs. not detected) and the Mann–Whitney U-test (levels) to adjust for incomplete parametric transformation with rank normalization. We performed multivariate logistic regression to identify chemicals that varied in level by clinical outcome (BPD, ROP), adjusting for potential confounders of clinical importance: birthweight, sex, corticosteroid treatment, type of nutrition (TPN vs. enteral nutrition) and maternal self-identified race/ethnicity. Logistic regression was performed in Python 3.8 using the statsmodel package. Analyses were performed within each study cohort separately, and results were combined in a meta-analysis using the metafor package in R 4.2.1 to account for study and experimental batch effects between the two cohorts (TOLSURF and PROP). All source code is available on GitHub. Metabolite data are presented as mean ± sd or as median and interquartile (IQ) range.

## Results

### Clinical Cohorts

The TOLSURF ( *n* = 171) and PROP ( *n* = 143) cohorts with metabolomic data were similar with regard to birthweight, sex, and distribution of maternally identified race/ethnicity. The two groups differed significantly ( *p* < 0.05) in terms of gestational age (TOLSURF mean 25.3 weeks and PROP 25.9 weeks), and PROP infants had less severe illness based on Respiratory Severity Score (FiO _2_ × mean airway pressure) and incidence of BPD and ROP. The mean age for collection of urines at time points 1 and 2 differed significantly between cohorts by 2.9 and 0.5 days, respectively ( Table 1 ).

**Table 1:** Characteristics of Infants included in cross-sectional metabolomic studies

	TOLSURF	PROP	*p* -Value
Number of Infants	171	143	–
Maternally identified Race/ethnicity	61|25|85	60|23|60	0.38
Black/Hispanic/White, ( *n* %)	(35.7/14.6/49.7)	(42.0,16.1/42.0)	
Gestational age (wk)	25.3 (1.2)	25.9 (1.2)	**<0.001**
Birth weight (g)	725 (175)	785 (150)	0.56
Male/female, *n* (%)	100/71 (58.5/41.5)	75/68 (52.4/47.6)	0.31
Average RSS days 7–14	4.2 (1.9)	2.6 (1.6)	**<0.001**
BPD at 36 weeks (yes/no), *n* (%)	106|65 (62.0/38.0)	72|71 (50.3/49.7)	**0.040**
ROP ≥stage 2 (yes/no), *n* (%)	105/66 (61.4/38.6)	63/80 (44.1/55.9)	**0.002**
Age urine sample 1	9.6 (2.4)	6.7 (1.1)	**<0.001**
Age urine sample 2	27.6 (2.2)	28.1 (1.0)	**0.006**

Abbreviations: BPD, bronchopulmonary dysplasia; ROP, retinopathy of prematurity.

Notes: Quantitative measurements mean (sd) were compared using the Student's *t* -test and categorical data using the Fisher Exact test. RSS, respiratory severity score (FiO _2_ × mean airway pressure).

*p* < 0.05 in bold.

### Detection and Relative Abundance of APAP Metabolites

Untargeted metabolomic analysis identified 233 named xenobiotic chemicals, including 9 APAP-related chemicals. Three metabolizing pathways for APAP (sulfation, glucuronidation, and oxidation (via CYP2e1) have been described. Our study detected unaltered APAP and a total of eight APAP metabolites in infant urine samples: four sulfated metabolites, two glucuronide metabolites, and two cysteinyl metabolites (via oxidation). Detection rates ranged from 8.1% (2-methoxyAPAP glucuronide) to 94.6% (4-APAP sulfate). 4-APAP sulfate was the most abundant urinary metabolite, based on relative values for area under the peak for each urine sample, with all other APAP metabolites at <15% ( Table 2 ).

**Table 2:** Detection and relative abundance of APAP metabolites in infant urine, cord plasma, and maternal plasma

	Infant urine		Cord plasma		Maternal plasma			
Chemical	Detect	Relative abundance	Detect	Relative abundance	Detect		Relative abundance	
	%	median (IQ)	%	mean (SD)	%	*p* vs. urine	mean (SD)	*p* vs. cord
4-APAP sulfate	94.6	1.00	75.0	1.00	58.3	**<0.001**	1.00	
3-(methylthio)APAP sulfate a	62.1	0.14 (0.07/0.39)						
3-(N-acetyl-cystein-S-yl)APAP	82.6	0.11 (0.07/0.17)	66.7	0.42 (0.51)	33.3	**0.05**	0.02 (0.01)	**0.005**
APAP	37.6	0.08 (0.04/0.13)	58.3	0.26 (0.06)	41.7	0.76	0.28 (0.05)	0.5
2-hydroxyAPAP sulfate a	20.2	0.11(0.07/0.12)	66.7	0.56 (0.50)	58.3	**<0.001**	0.40 (0.19)	0.52
2-methoxyAPAP sulfate a	46.3	0.04 (0.07/0.35)	33.3	0.02 (0.01)	25.0	0.18	0.02 (0.02)	0.93
3-(cystein-S-yl)APAP a	29.9	0.03 (0.02/0.06)	50.0	0.59 (0.38)	41.7	**0.03**	0.21 (0.11)	0.06
4-APAP glucuronide	40.9	0.02 (0.01/0.04)	66.7	1.56 (0.92)	50.0	0.53	0.90 (0.49)	0.17
2-methoxyAPAP glucuronide a	8.1	0.003 (0.002/0.01)	41.7	0.04 (0.05)	33.3	**<0.001**	0.04 (0.02)	0.81

Abbreviation: APAP, acetaminophen.

identity.

Notes: Abundance was calculated from the area under the peak for each sample and normalized to the 4-acetaminophen sulfate area in the same sample.

*n* = 171 TOLSURF plus 143 PROP infants and 628 total urines collected at days 10 and 28.

Cord: *n* = 12 infants for detection rate, 4–9 for relative abundance.

Maternal: *n* = 12 for detection rate, 3–7 for relative abundance.

*p* ≤ 0.05 shown in bold; % detected by chi-squared test; relative abundance by unpaired *t* -test.

a Chemical that has not been confirmed based on a standard, but Metabolon is confident in its identity.

**Fig. 1:**
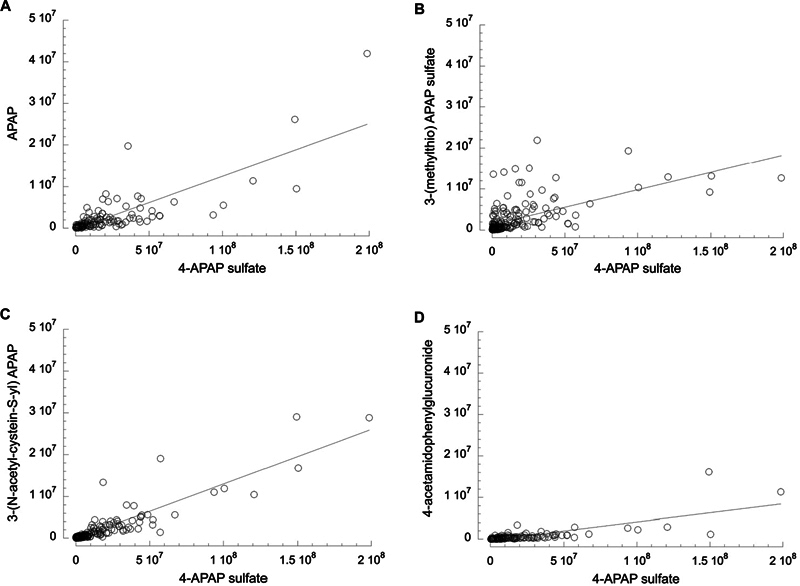
Comparison of abundance between selected urinary APAP metabolites. ( **A** ) 4-APAP sulfate versus APAP; *r* = 0.79, *p* = 3.6e-28, *n* = 129. ( **B** ) 4-APAP sulfate versus 3-(methylthio) APAP sulfate; *r* = 0.59, *p* = 2.9e-20, *n* = 203. ( **C** ) 4-APAP sulfate versus 3-(N-acetyl-cystein-S-yl) APAP; *r* = 0.90, *p* = 7e-108, *n* = 296. ( **D** ) 4-APAP sulfate versus 4-acetamidophenylglucuronide; *r* = 0.75, *p* = 5.1e-26, *n* = 140. Data are unadjusted area under the curve values from MS:MS for urines where both metabolites were detected and quantified in the sample. Analysis by linear regression with n values as listed. Note the fourfold difference in scale between the vertical and horizontal axis. APAP, acetaminophen.

There were high correlations between levels of 4-APAP sulfate and the other metabolites by linear regression with r values ranging from 0.58 (2-hydroxyAPAP sulfate) to 0.90 (APAP) with all *p* -values <0.0003. Fig. 1 shows four examples of regression plots for 4-APAP sulfate versus other APAP metabolites in the same sample, illustrating high correlations between 4-APAP-sulfate and APAP ( Fig. 1A ), another sulfate derivative ( Fig. 1B ), a cysteinyl derivative ( Fig. 1C ) and a glucuronide derivative ( Fig. 1D ).

To assess the exposure of infants to APAP before birth, we examined the detection rate and relative abundance of metabolites in cord and maternal plasma samples from a separate cohort of premature infants using the same metabolomic platform (Metabolon Inc) for analysis ( Table 2 ). The range of detection rates for APAP and seven metabolites were similar for cord (33–75% of infants) and maternal samples (25–58% of mothers). The abundance of all APAP metabolites (relative to 4-APAP sulfate) was similar in cord and maternal plasma except for the two cysteinyl derivatives that were lower in fetal plasma. Compared with infant urine, detection rates for maternal plasma were significantly higher for three metabolites: 2-hydroxyAPAP sulfate (58% maternal vs. 12% urine), 3-(cystein-S-yl)APAP (42% vs. 17%) and 2-methoxyAPAP glucuronide (33% vs. 8%), and lower for two metabolites: 4- APAP sulfate and 3-(N-acetyl-cystein-yl) APAP. There was a high relative abundance of 4-APAPglucuronide in maternal (0.90) and cord plasma (1.56) vs. urine (0.02, which is consistent with increased glucuronidation during postnatal development. 30 31 32

### APAP Levels in Time Course Studies

**Fig. 2:**
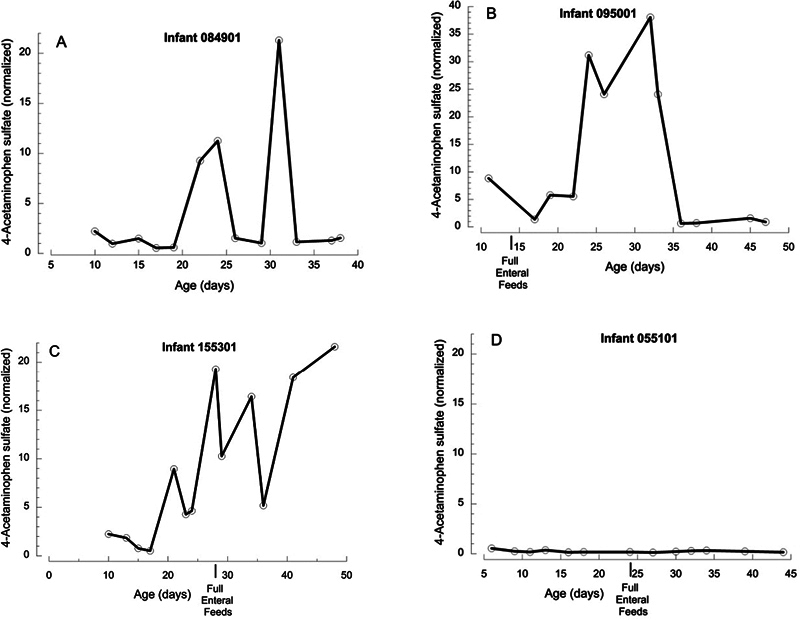
Time course for levels of urinary 4-APAP sulfate in four infants. ( **A** ) 25.6-week gestation infant with BPD, ROP, and PDA who remained on TPN until day 46 with two episodes of elevated metabolite (values > 2). ( **B** ) 25.1-week gestation infant with ROP who transitioned to full enteral feeds on day 14; 4-APAP sulfate was elevated at day 10 and between day 18 and 33 (note the higher scale of the *x* -axis). ( **C** ) 25.0-week gestation infant with BPD and ROP on full enteral feeds from day 28; 4-APAP sulfate was elevated from day 21 to 48. ( **D** ) 24.1-week gestation infant with BPD and ROP on full enteral nutrition from day 24; all values are less than 1. APAP, acetaminophen.

For time course studies in a subset of 28 TOLSURF infants, we used 4-APAP sulfate as a representative urinary indicator of APAP exposure based on the high detection rate and strong correlations of levels with those of other derivatives. In serial collections of urine, most (23/28, 82%) TOLSURF infants had two or more consecutive values of 4-APAP sulfate >two-fold of the median (defined as peaks). These occurred in six infants only during TPN, six infants only while on enteral feeds, and 11 infants during both time periods. Time course plots for four infants ( Fig. 2 ) show examples of periods of elevated urinary 4-APAP sulfate during TPN (panel a), while on full enteral nutrition (panels b), and during both modes of nutrition (c) compared with an infant with levels <1.0 in all samples (representative of five infants, panel d), illustrating the episodic nature of increased APAP levels imposed on a lower baseline concentration.

**Fig. 3:**
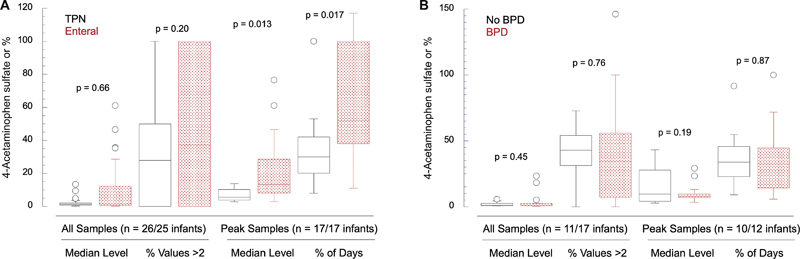
Summary of time course parameters for infants with longitudinal data. ( **A** ) 4-APAP sulfate levels in infant urine during TPN ( *n* = 26 infants, 151 samples) or enteral nutrition ( *n* = 25 infants, 142 samples); data summarize results for 4-APAP sulfate levels in all infants (comparisons 1 and 2, *n* = 25 and 26 infants during TPN and enteral nutrition, respectively) and for those infants with levels >2 (comparisons 3–4, *n* = 17 and 17). Both the median level of 4-APAP sulfate peaks and the duration of peaks are ∼three-fold greater on enteral nutrition ( *p* ≤ 0.01). ( **B** ) Summary of same-time course parameters for 4-APAP levels in infants with BPD ( *n* = 17 infants) versus those without BPD ( *n* = 11); there are no significant differences. Values >2 (elevated values) are defined as more than twice the median value. Peaks are defined as two or more consecutive urine samples with values >2. The interval of urine collections for infants with peaks was similar during TPN and enteral nutrition (15.3 ± 9.7 and 17.4 ± 9.2 days, respectively). APAP, acetaminophen.

Because of the report associating levels of APAP in breast milk with BPD and ROP, 17 we further explored urine APAP levels related to the source of nutrition. We separately examined APAP levels from samples collected during TPN and after the transition to enteral nutrition with breast milk ( Fig. 3A ); the median postnatal age of urine collections after transitioning to enteral nutrition was 28 days (range 11–63) compared with 16 days (range 6–41, *p* < 0.001) during TPN nutrition. For all time course samples (TPN *n* = 151; enteral *n* = 142), both levels of 4-APAP sulfate ( Fig. 3A , comparison 1), and % of values >2 ( Fig. 3A , comparison 2) were not statistically different by feeding status (TPN vs. enteral). During the intervals of urine collection, 17 infants in each nutritional group had episodes of elevated values (peaks). In these infants with peaks of 4-APAP sulfate, the level was higher during enteral (median 13.3) than during TPN feeds (median 5.8, Fig. 3A , comparison 3). In addition, the duration of peaks was longer during enteral than TPN feeds expressed as both total days (10.5 ± 7.1 days and 4.2 ± 2.0 days, respectively, *p* = 0.014) and as percent of total days of urine collection ( Fig. 3A , comparison 4).

Comparing the same four time-course parameters between infants with ( *n* = 17) and without ( *n* = 11) BPD revealed no significant differences ( Fig. 3B ). Similarly, there were no differences detected examining ROP as the outcome (data not shown). Thus, all infants in the longitudinal analysis group had detectable levels of APAP metabolite at all collection times during the first two postnatal months, and most infants had one or more periods of elevated levels. These episodes had higher levels and were longer in duration later in the postnatal course while receiving enteral nutrition. However, there were no significant associations of elevated levels with the two clinical outcomes.

### APAP Metabolite Levels in Cross-Sectional Analyses

Urinary levels of 4-APAP sulfate varied markedly between 314 infants at both day 10 and 28 (2,163- and 15,692-fold, respectively), which is consistent with intermittent exposure to higher doses of APAP during the first postnatal month. For all samples, there was no difference by time point (day 10 vs. 28) in number of infants with 4-APAP sulfate values >two-fold above the median value (39.2 vs. 37.0%), nor in median values of levels >2 (8.6 and 9.7, respectively). In day-28 samples, the number of infants with values >2 was greater for infants on enteral versus parenteral nutrition (52.4% vs. 25.5%, *p* < 0.0001, respectively), and median levels were similar in both groups (10.6 vs. 9.5, respectively).

Using multivariate logistic regression and meta-analysis of the two infant cohorts, we examined the cross-sectional metabolomic data on days 10 and 28 for associations between clinical outcomes and levels of APAP and all detected APAP metabolites. BPD, which was defined as a need for positive pressure or supplemental oxygen at 36 weeks, occurred in 56.7% of the 314 study infants, and ROP of stage ≥2 occurred in 53.9%. Analyses at day 28 also were performed separately for infants on TPN versus full enteral nutrition because of the marked effect of feeding status on the infant metabolome 26 and because of the reported associations between APAP levels in breast milk and premature infant outcomes. 17

In both day-10 and day-28 urine samples there were no significant associations ( *p* < 0.05) between detection rate or levels of APAP metabolites and BPD by univariate analyses (data not shown). In multivariate logistic regression of APAP metabolite levels, adjusting for birth weight, sex, corticosteroid treatment, type of nutrition (TPN vs. enteral nutrition), and maternal self-identified race/ethnicity, no significant associations were found for all samples at day 10 ( Table 3 , data columns, 1–6) or at day 28 (columns, 7–12).

**Table 3:** Association of urinary APAP metabolites with BPD by multivariate logistic regression and meta-analysis for all samples

	TOLSURF ( *n* = 171)		PROP ( *n* = 143)		Meta ( *n* = 314)		TOLSURF ( *n* = 171)		PROP ( *n* = 143)		Meta ( *n* = 314)	
Chemical	All values on day 10						All values on day 28					
	OR (CI)	*p*	OR (CI)	*p*	OR (CI)	*p*	OR (CI)	*p*	OR (CI)	*p*	OR (CI)	*p*
4-APAP sulfate	1.00 (0.98/1.01)	0.69	1.02 (0.98/1.05)	0.44	1.00 (0.98/1.02)	0.98	1.00 (0.99/1.01)	0.79	0.99 (0.98/1.01)	0.41	1.00 (0.99/1.01)	0.91
3-(methylthio)APAP sulfate a	1.01 (0.91/1.12)	0.82	0.99 (0.87/1.14)	0.94	1.00 (0.93/1.09)	0.89	0.97 (0.89/1.06)	0.53	0.96 (0.91/1.02)	0.19	1.00 (0.96/1.04)	0.96
3-(N-acetyl-cystein-S-yl) APAP	1.00 (0.98/1.02)	0.95	1.03 (0.97/1.09)	0.31	1.00 (0.98/1.03)	0.67	1.00 (0.99/1.02)	0.68	1.00 (0.99/1.00)	0.53	1.00 (0.99/1.00)	0.80
APAP	0.93 (0.83/1.04)	0.21	1.09 (0.86/1.38)	0.49	0.96 (0.86/1.06)	0.41	1.06 (0.87/1.29)	0.54	0.98 (0.92/1.04)	0.47	1.00 (0.96/1.04)	0.97
2-hydroxyAPAP sulfate a	1.06 (0.45/2.50)	0.90	1.15 (0.77/1.71)	0.49	1.13 (0.79/1.62)	0.50	1.00 (0.75/1.32)	0.98	0.86 (0.69/1.06)	0.15	0.99 (0.94/1.04)	0.71
2-methoxyAPAP sulfate a	0.97 (0.86/1.09)	0.58	1.02 (0.90/1.16)	0.73	0.99 (0.91/1.08)	0.87	1.01 (0.95/1.08)	0.68	0.99 (0.98/1.01)	0.47	1.00 (0.99/1.01)	0.91
3-(cystein-S-yl)APAP a	1.06 (0.80/1.40)	0.69	1.10 (0.94/1.29)	0.23	1.09 (0.94/1.25)	0.22	1.01 (0.92/1.10)	0.76	1.00 (0.97/1.02)	0.75	1.00 (0.99/1.00)	0.68
4-APAP glucuronide	1.03 (0.92/1.16)	0.57	1.08 (0.82/1.41)	0.59	1.04 (0.94/1.16)	0.46	1.01 (0.95/1.08)	0.70	0.99 (0.93/1.06)	0.83	1.00 (0.99/1.01)	0.68
2-methoxyAPAP glucuronide a	1.06 (0.52/2.18)	0.87			1.06 (0.52/2.18)	0.87	1.10 (0.83/1.45)	0.53	0.93 (0.67/1.29)	0.66	0.99 (0.95/1.04)	0.67

Abbreviations: APAP, acetaminophen; CI, confidence interval; OR, odds ratio.

Note: Meta: A meta-analysis of logistic regression results from TOLSURF plus PROP.

a Chemical that has not been confirmed based on a standard, but Metabolon is confident in its identity.

We also examined APAP associations with BPD and ROP by multivariate logistic regression in day-28 infants separately by nutritional source: TPN = 188 infants (55.9% BPD); enteral nutrition = 126 infants (57.9% BPD). By meta-analysis, there were no significant associations for infants on TPN ( Table 4 , data columns 1–6) and one significant association for infants on enteral nutrition (columns, 7–12): 3-(methylthio)APAP sulfate (OR = 0.91, CI: 0.82/1.00, *p* = 0.045, last column), indicating lower metabolite levels in infants with BPD. OR values for the other metabolites on enteral nutrition were all <1 with insignificant *p* -values ranging from 0.07 to 0.76 in the cross-cohort meta-analysis. In the TOLSURF cohort on enteral nutrition (columns, 19 and 20), six metabolites had *p* < 0.05 and OR <1 consistent with decreased risk of BPD, but statistical significance was not observed for the PROP cohort. The same analyses were performed for ROP, and there were no significant associations of APAP metabolites at either time point ( Table 5 ) or by feeding status ( Table 6 ). Thus, levels of APAP urinary metabolites were not associated with increased risk for two outcomes that are diagnosed later in the clinical course after exposure to APAP.

**Table 4:** Association of urinary APAP metabolites with BPD by feeding status at day 28

	TOLSURF ( *n* = 108)		PROP ( *n* = 80)		Meta ( *n* = 188)		TOLSURF ( *n* = 63)		PROP ( *n* = 63)		Meta ( *n* = 126)	
Chemical	Infants on TPN						Infants on enteral nutrition					
	OR (CI)	*p*	OR (CI)	*p*	OR (CI)	*p*	OR (CI)	*p*	OR (CI)	*p*	OR (CI)	*p*
4-APAP sulfate	1.01 (0.98/1.04)	0.45	0.99 (0.98/1.01)	0.45	0.98 (0.99/1.01)	0.69	0.92 (0.97/0.98)	**0.009**	0.99 (0.96/1.01)	0.40	0.98 (0.95/1.00)	0.066
3-(methylthio)APAP sulfate a	1.11 (0.95/1.30)	0.20	0.97 (0.92/1.03)	0.37	0.99 (0.94/1.04)	0.68	0.82 (0.67/1.00)	0.055	0.93 (0.83/1.04)	0.22	0.91 (0.82/1.00)	**0.045**
3-(N-acetyl-cystein-S-yl)APAP	1.03 (0.98/1.09)	0.26	1.00 (0.99/1.00)	0.47	1.00 (0.99/1.00)	0.53	0.90 (0.82/0.98)	**0.017**	1.00 (0.98/1.01)	0.71	0.99 (0.92/1.01)	0.37
APAP	1.25 (0.82/1.91)	0.30	0.97 (0.91/1.04)	0.41	0.98 (0.92/1.05)	0.51	0.63 (0.35/1.10)	0.106	0.98 (0.82/1.17)	0.80	0.94 (0.79/1.11)	0.47
2-hydroxyAPAP sulfate a	2.42 (0.42/14.1)	0.33	0.90 (0.68/1.20)	0.47	0.92 (0.70/1.22)	0.58	0.03 (0.01/0.68)	**0.028**	0.78 (0.55/1.11)	0.16	0.75 (0.53/1.06)	0.11
2-methoxyAPAP sulfate a	1.29 (0.92/1.81)	0.14	0.99 (0.98/1.01)	0.46	0.99 (0.98/1.01)	0.50	0.72 (0.54/0.94)	**0.017**	0.96 (0.89/1.04)	0.32	0.94 (0.88/1.01)	0.11
3-(cystein-S-yl)APAP a	1.30 (0.67/2.54)	0.44	0.99 (0.96/1.03)	0.77	0.99 (0.96/1.04)	0.80	0.37 (0.14/0.97)	**0.043**	1.00 (0.98/1.03)	0.81	1.00 (0.97/1.03)	0.76
4-APAP glucuronide	1.11 (0.90/1.35)	0.33	1.00 (0.98/1.02)	0.70	1.00 (0.98/1.02)	0.77	0.42 (0.18/0.96)	**0.040**	0.89 (0.73/1.09)	0.25	0.85 (0.70/1.04)	0.11
2-methoxyAPAP glucuronide a			0.98 (0.90/1.06)	0.62	0.98 (0.87/1.06)	0.62			0.83 (0.50/1.89)	0.49	0.79 (0.48/1.32)	0.36

Abbreviations: APAP, acetaminophen; CI, confidence interval; OR, odds ratio.

Notes: Meta: A meta-analysis of logistic regression results from TOLSURF plus PROP.

*p* -Values ≤0.05 in bold.

a Chemical that has not been confirmed based on a standard, but Metabolon is confident in its identity.

**Table 5:** Association of urinary APAP metabolites with ROP by multivariate logistic regression and meta-analysis

	TOLSURF ( *n* = 171)		PROP ( *n* = 143)		Meta ( *n* = 314)		TOLSURF ( *n* = 171)		PROP ( *n* = 143)		Meta ( *n* = 314)	
Chemical	All values on day 10						All values on day 28					
	OR (CI)	*p*	OR (CI)	*p*	OR (CI)	*p*	OR (CI)	*p*	OR (CI)	*p*	OR (CI)	*p*
4-APAP sulfate	0.99 (0.98/1.01)	0.56	0.99 (0.94/1.02)	0.27	0.99 (0.98/1.01)	0.32	1.00 (0.99/1.00)	0.24	0.98 (0.96/1.00)	0.10	1.00 (0.99/1.00)	0.13
3-(methylthio)APAP sulfate a	0.98 (0.89/1.07)	0.69	0.89 (0.76/1.04)	0.14	0.95 (0.88/1.04)	0.27	1.02 (0.93/1.13)	0.62	0.96 (0.90/1.02)	0.20	0.98 (0.93/1.03)	0.43
3-(N-acetyl-cystein-S-yl)APAP	0.99 (0.97/1.01)	0.49	0.96 (0.90/1.03)	0.24	0.99 (0.97/1.01)	0.30	1.00 (0.99/1.00)	0.23	0.96 (0.91/1.01)	0.12	0.99 (0.99/1.00)	0.16
APAP	0.94 (0.84/1/06)	0.31	0.91 (0.71/1.16)	0.44	0.94 (0.84/1.04)	0.21	0.98 (0.94/1.02)	0.24	0.90 (0.79/1.03)	0.13	0.97 (0.93/1.01)	0.12
2-hydroxyAPAP sulfate a	1.00 (0.42/2.39)	0.99	0.89 (0.58/1.36)	0.59	0.91 (0.62/1.33)	0.63	0.84 (0.63/1.12)	0.23	0.83 (0.66/1.05)	0.12	0.83 (0.70/1.00)	**0.05**
2-methoxyAPAP sulfate a	1.03 (0.92/1.15)	0.65	0.92 (0.80/1.05)	0.23	0.98 (0.90/1.07)	0.67	0.99 (0.96/1.10)	0.24	0.95 (0.88/1.02)	0.19	0.98 (0.96/1.01)	0.13
3-(cystein-S-yl)APAP a	0.92 (0.71/1.20)	0.55	0.92 (0.79/1.07)	0.27	0.92 (0.80/1.05)	0.21	0.96 (0.89/1.03)	0.25	0.94 (0.87/1.01)	0.10	0.95 (0.90/1.00)	**0.05**
4-APAP glucuronide	0.96 (0.89/1.05)	0.38	0.91 (0.69/1.20)	0.49	0.96 (0.89/1.04)	0.30	0.99 (0.97/1.01)	0.25	0.90 (0.77/1.05)	0.18	0.99 (0.96/1.01)	0.19
2-methoxyAPAP glucuronide a	1.58 (0.58/4.29)	0.37			1.58 (0.58/4.29)	0.37	0.95 (0.86/1.05)	0.35	0.72 (0.44/1.18)	0.20	0.94 (0.86/1.04)	0.24

Abbreviations: APAP, acetaminophen; CI, confidence interval; OR, odds ratio.

Notes: Meta: A meta-analysis of logistic regression results from TOLSURF plus PROP.

*p* -Values ≤0.05 in bold.

a Chemical that has not been confirmed based on a standard, but Metabolon is confident in its identity.

**Table 6:** Association of urinary APAP metabolites with ROP by feeding status at day 28

	TOLSURF ( *n* = 108)		PROP ( *n* = 80)		Meta ( *n* = 188)		TOLSURF ( *n* = 63)		PROP ( *n* = 63)		Meta ( *n* = 126)	
Chemical	Infants on TPN day 28						Infants on enteral nutrition day 28					
	OR (CI)	*p*	OR (CI)	*p*	OR (CI)	*p*	OR (CI)	*p*	OR (CI)	*p*	OR (CI)	*p*
4-APAP sulfate	1.00 (0.99/1.00)	0.23	0.97 (0.91/1.03)	0.27	0.99 (0.99/1.00)	0.18	1.03 (0.98/1.09)	0.24	0.99 (0.96/1.02)	0.39	1.00 (0.97/1.02)	0.86
3-(methylthio)APAP sulfate a	1.04 (0.90/1.20)	0.57	0.94 (0.86/1.03)	0.19	0.97 (0.90/1.05)	0.42	1.01 (0.89/1.15)	0.88	0.98 (0.89/1.08)	0.71	0.99 (0.92/1.07)	0.84
3-(N-acetyl-cystein-S-yl)APAP	1.00 (0.99/1.00)	0.23	0.92 (0.82/1.02)	0.12	1.00 (0.99/1.00)	0.19	1.01 (0.95/1.07)	0.82	0.98 (0.94/1.02)	0.27	0.99 (0.95/1.02)	0.43
APAP	0.98 (0.93/1.02)	0.24	0.72 (0.37/1.37)	0.32	0.97 (0.93/1.02)	0.22	1.21 (0.70/2.06)	0.50	0.97 (0.80/1.17)	0.74	0.99 (0.83/1.18)	0.93
2-hydroxyAPAP sulfate a	0.79 (0.51/1.21)	0.27	0.66 (0.41/1.08)	0.10	0.73 (0.53/1.01)	**0.05**	2.44 (0.44/13.42)	0.31	1.04 (0.75/1.45)	0.82	1.07 (0.77/1.48)	0.68
2-methoxyAPAP sulfate a	0.98 (0.96/1.01)	0.24	0.90 (0.75/1.08)	0.26	0.98 (0.96/1.01)	0.19	1.04 (0.86/1.27)	0.67	0.97 (0.89/1.05)	0.42	0.98 (0.90/1.06)	0.57
3-(cystein-S-yl)APAP a	0.95 (0.88/1.04)	0.28	0.85 (0.64/1.13)	0.27	0.94 (0.87/1.03)	0.17	1.16 (0.68/1.05)	0.59	0.96 (0.88/1.03)	0.24	0.96 (0.89/1.04)	0.28
4-APAP glucuronide	0.99 (0.96/1.01)	0.25	0.79 (0.57/1.11)	0.18	0.99 (0.96/1.01)	0.22	1.05 (0.79/1.38)	0.76	0.96 (0.78/1.18)	0.70	0.99 (0.84/1.17)	0.90
2-methoxyAPAP glucuronide a	0.95 (0.85/1.05)	0.28	1.13 (1.01/1.88)	0.14	0.94 (0.85/1.04)	0.26	1.04 (0.86/1.27)	0.67	0.97 (0.89/1.05)	0.42	0.98 (0.90/1.06)	0.57

Abbreviations: APAP, acetaminophen; CI, confidence interval; OR, odds ratio.

Note: Meta: A meta-analysis of logistic regression results from TOLSURF plus PROP.

*p* -Values ≤0.05 in bold.

a Chemical that has not been confirmed based on a standard, but Metabolon is confident in its identity.

## Discussion

In this study, we used untargeted metabolomics to examine urinary metabolites of APAP and their associations with clinical outcomes in extremely low gestation premature infants. The major metabolite 4-APAP sulfate was detected in 98% of urine samples collected between 6 and 59 days; most infants had intervals of elevated levels that were higher and of longer duration while on enteral feeds. At both 10 and 28 days, levels of APAP and eight APAP metabolites were not significantly associated with BPD or ROP, providing evidence derived from the infant urinary metabolome that APAP exposure is not associated with these adverse clinical outcomes.

As reviewed, there has been concern regarding the safety of APAP in infants based on animal data, individual pharmacokinetic data, known toxicity at higher doses in adults, and results from some non-randomized clinical studies in infants. 12 13 In particular, the safety of APAP has not been established in clinical trials with extremely premature infants. Of concern, in a recent metabolomic study with infants of 26 weeks mean gestational age, Santoro et al 17 reported significant associations between levels of APAP and four APAP metabolites in maternal breast milk with BPD ( *p* -values 0.009–0.04 and OR values, 3.1–6.7) and between APAP and ROP ( *p* = 0.04 and OR = 2.7). Our study in infant urine does not confirm this association; in fact, most OR values for APAP metabolites and BPD were <1, including an association by meta-analysis at *p* = 0.05 for 3-(methylthio)APAP sulfate, suggesting possible protection by APAP.

Both the Santoro and our study were conducted in extremely low gestation newborns of similar gestational and birth weight (breast milk/urine mean gestational age 26.3/25.3 weeks and mean birth weight 840/727 g, respectively) who were born in the same era (breast milk 2009–2011, urine 2010–2013) and used the same metabolomics platform (Metabolon), which provided consistency for the analytic approach and metabolite library. In both studies, convenience samples were used for the analysis. Our definition of BPD (physiologic requirement for supplemental oxygen and/or positive pressure) was somewhat more rigorous than in the Santoro study, which may account for the somewhat lower incidence of BPD in our cohorts (TOLSURF = 62.0%, PROP = 50.3%) compared with Santoro (65.3%). The most important difference between the two studies was the measurement of APAP in the infant urine versus in maternal breast milk, which may not necessarily reflect APAP levels in the infant because of variable milk consumption and different relative abundance of metabolites after maternal metabolism. The parallel APAP association results for BPD and ROP, in both the Santoro study and ours, are consistent with these disorders tending to occur in the same, sicker infants. We believe that our results provide strong evidence for the lack of a clinically significant association between APAP exposure, at the levels experienced by TOLSURF and PROP infants, and both BPD and ROP. However, a definitive conclusion may require an additional study with measurements in both breast milk and in the infant.

Premature infants can be exposed to APAP in utero, by direct administration in the NICU, and via breast milk secondary to maternal intake. Because APAP is considered safe (up to 3 g per day) during pregnancy, many infants are exposed in utero intermittently throughout gestation, including during labor both preterm and at term. Our studies with cord plasma found 4-APAP sulfate in 75% of preterm infants at birth (and their mothers) with a similar detection rate in term infants (data not presented); this is consistent with the reported use of APAP in pregnancy. 33 34 With a half-life of approximately 4 hours in infants, APAP, and its metabolites should be fully cleared after a few days, and thus prenatal exposure does not explain the nearly uniform presence of 4-APAP sulfate in infant urines collected at 7 to 14 days.

In a large hospital network, APAP was number 10 of the top 100 drugs used in NICUs, and exposure increased between 2010 and 2018. 1 In the UCSF NICU, one of the sites for both studies, APAP is routinely prescribed as an analgesic for infant pain/discomfort on an as-needed basis at approximately 10 mg/kg q 6 hours. Data for postnatal administration of APAP were not recorded in either TOLSURF or PROP, and therefore we could not evaluate APAP metabolite levels as related to frequency and dose of treatment. Based on the quality control measures during metabolomic analysis, and the absence of APAP metabolites in saline exposed to cotton balls on a diaper (Methods), the high detection rate for 4-APAP sulfate is unlikely to be a technical artifact. Our findings of detectable urinary 4-APAP sulfate in most urine samples from infants (up to 46 days postnatal age) may reflect frequent use of APAP for low-level pain at many of the study sites as a possible explanation for baseline urinary levels, but not episodic increases. The higher occurrence rate and levels of 4-APAP sulfate compared with APAP may indicate a slower clearance for the sulfate metabolite; however, there is no pharmacokinetic data on this topic. Clearance of APAP is slower in extremely premature infants compared with more mature, older infants, which may also contribute to the high detection rate of 4-APAP sulfate. 35

Following the report by Hammerman et al in 2011, 36 APAP has increasingly been used to promote closure of the PDA, initially as a backup drug to indomethacin and ibuprofen and currently often as the front-line drug, and closure rate is serum level-dependent. 37 Use of APAP for PDA closure was not collected in TOLSURF or PROP; however, it is likely that some of the infants enrolled between 2012 and 2013 received APAP (at higher doses of 10–20 mg/kg q 6 hours with adjustment of dose based on plasma levels in some units). The highest levels of 4-APAP sulfate were observed in infants (18/19) with samples collected while on enteral feeds, and elevated levels were episodic rather than continuous in most infants. Most of these samples were collected beyond the age when PDA is usually treated, and we speculate that some of these elevated levels reflect APAP treatment for painful procedures.

The findings of Santoro et al 17 that 4-APAP sulfate was detected in 71% of breast milk samples at postnatal day 14 after preterm birth indicate that maternal treatment likely contributes to urinary APAP in infants on enteral feeds. In an earlier study with lactating women of term pregnancies, one dose of APAP resulted in similar levels of APAP in milk and plasma with peak levels of ∼0.01 mg/mL. 38 In several studies examining APAP levels in breast milk, it was calculated that term infants on breast milk would receive only 1.1–3.6% of the weight-adjusted daily recommended dose for adults. 39 Thus, maternal APAP likely does not explain episodes of high infant levels, although it is possible that some mothers of premature infants exceeded the recommended dose of APAP, leading to higher levels transferred to infants via breast milk. Additional pharmacokinetic studies of APAP and metabolites in extremely low gestation newborns, particularly in relationship to breast milk, are needed.

The hepatotoxicity associated with high APAP doses is well established and results from production by CYP2E1 of the metabolite N-acetyl-p-benzyl quinone; this toxic metabolite was not analyzed in our study; however, we did find low levels of APAP cysteinyl derivatives that result from binding of N-acetyl-p-benzyl quinone to cysteine in proteins. Cysteinyl derivatives are also found in the serum of most adults taking APAP at therapeutic doses at concentrations below the threshold associated with hepatotoxicity. 40 CYP2E1 is also present in the lung and may contribute to the reported association between APAP and chronic obstructive lung disease and asthma. 9 10 11 Because N-acetyl-p-benzyl quinone is inactivated by glutathione, toxic effects would presumably be greater under oxidative conditions with depletion of glutathione such as occurs in infants with lung disease who experience oxidative stress. This could lead to impaired alveolization in the developing lung and an increased likelihood of BPD. However, evidence for a causal link between APAP and BPD remains lacking.

Strengths of our study include the relatively large number of infants studied, the multi-institutional cohorts, the detailed clinical database, and both cross-sectional and longitudinal analyses; however, there are limitations. Some aspects of clinical care varied between institutions, and information on dose level and schedule of APAP treatment was not collected. Because the use of APAP for ductal closure has increased since 2013, exposure to higher doses of APAP is likely greater now than in our studies. Because data for APAP treatment of infants were not available, it was not possible to evaluate pharmacokinetics in the longitudinal study. Interpretation of elevated APAP metabolite results in the time course studies related to the nutritional source is complicated by the limited number of infants and older postnatal age of those on enteral nutrition. Although clinical data were not collected in TOLSURF for the type of milk (breast vs. formula) at the initiation of enteral feeds, we assume that most if not all infants received full or partial breast milk feeds. This was the standard of care in the NICU in 2010–2013 in compliance with the guidelines of the American Academy of Pediatrics. 41 Our previous metabolomic analysis of urines from infants on TPN versus enteral nutrition also supports this conclusion: in day-28 samples, levels of 45/69 chemicals in the Food Component subpathway of Xenobiotic chemicals were significantly ( *p* < 0.05) different by feeding status, and levels were higher for infants on enteral feeds for 91.1% of these metabolites. An example is cinnamoylglycine, the glycine derivative of cinnamic acid that is abundant in fruits and vegetables. The mean level of enteral nutrition was sixfold higher ( *p* < 0.001) than for infants on TPN. 26 The number of infants with cord blood that were studied was small, and this topic needs further investigation in a larger, contemporary cohort. Finally, we cannot rule out the possibility that confounding variables that were not assessed contribute to the lack of observed associations of outcomes with APAP metabolites.

## Conclusion

In conclusion, the major APAP metabolite was detected in most urine samples of all infants, and intervals of elevated levels occurred that were higher and of longer duration during enteral nutrition. Using both longitudinal and cross-sectional analyses at two-time points, we found no association in two infant cohorts between APAP levels and BPD or ROP. Although APAP is known to have toxic effects at high doses, our results suggest that APAP exposure, at doses experienced by infants in our cohorts, does not increase the risk for two adverse outcomes in the neonatal period. Importantly, our data do not address the likely increased risks associated with prolonged exposure to higher doses in premature infants, and the results need confirmation by examining total APAP exposure over time.
